# Supplementary material for: Prediction and verification of the AD-FTLD common pathomechanism based on dynamic molecular network analysis
Source: Commun Biol. 2021 Aug 12;4:961. doi: 10.1038/s42003-021-02475-6 (PMC8361101; doi:10.1038/s42003-021-02475-6)

Supplementary Information

Prediction and verification of the AD-FTLD common pathomechanism based on dynamic molecular network analysis

Meihua Jin[1,#], Xiaocen Jin[1,#], Hidenori Homma[1,#, $], Kyota Fujita[1], Hikari Tanaka[1], Shigeo Murayama[2], Hiroyasu Akatsu[3], Kazuhiko Tagawa[1] and Hitoshi Okazawa[1,4, $]

1: Department of Neuropathology, Medical Research Institute, Tokyo Medical and Dental University, 1-5-45, Yushima, Bunkyo-ku, Tokyo 113-8510, Japan

2: Department of Neuropathology, Brain Bank for Aging Research, Tokyo Metropolitan Institute of Gerontology, 35-2, Sakae-cho, Itabashi-ku, Tokyo 173-0015, Japan

3: Department of Medicine for Aging in Place and Community-Based Medical Education, Nagoya City University Graduate School of Medical Sciences, Nagoya, Aichi 467-8601, Japan

4: Center for Brain Integration Research, Tokyo Medical and Dental University, 1-5-45, Yushima, Bunkyo-ku, Tokyo 113-8510, Japan

#: Equally contributed

$: Correspondence should be addressed to H.O and H.H..

E-mail: okazawa-tky@umin.ac.jp

Supplementary Figure 1

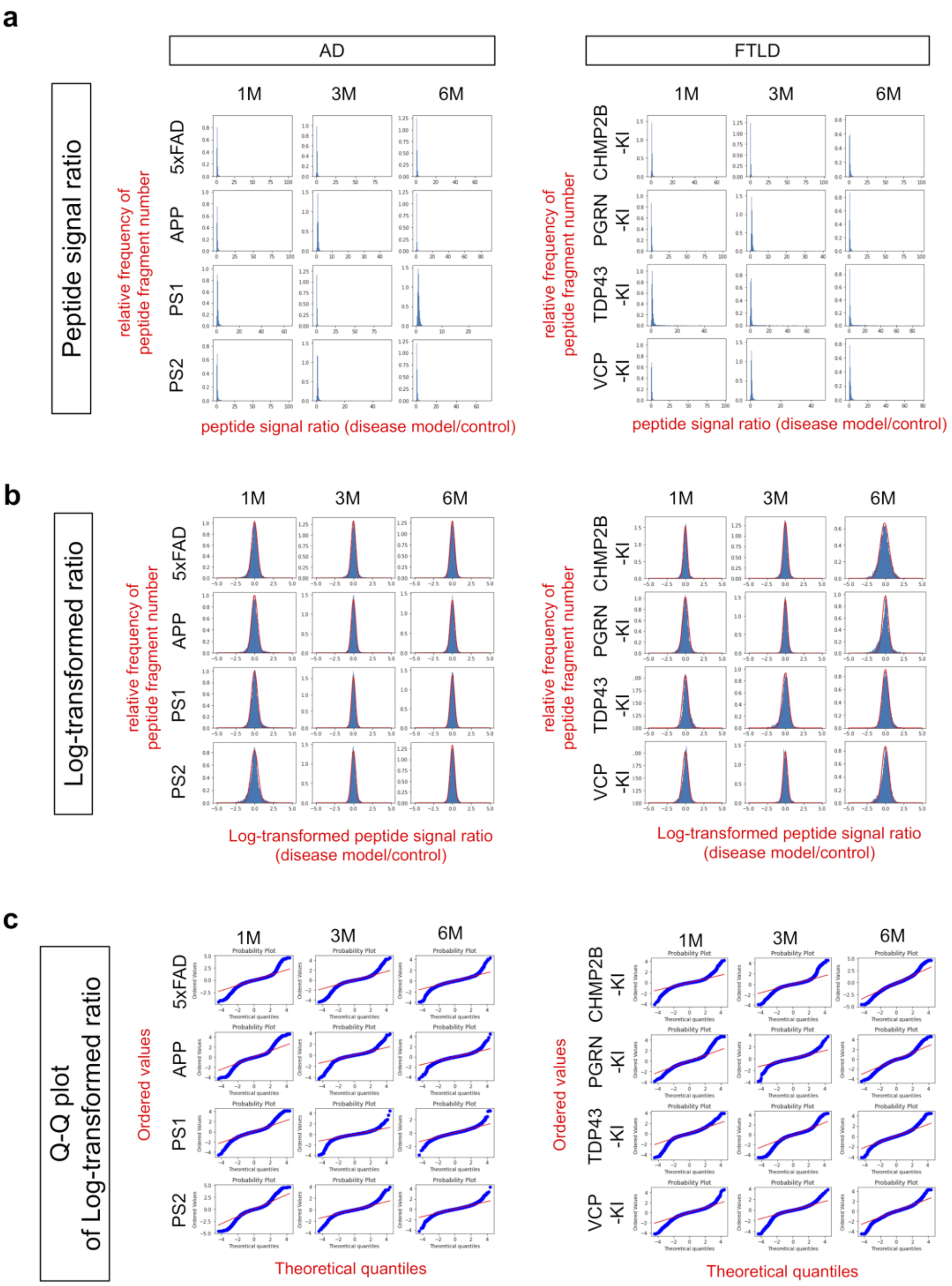

Supplementary Figure 1
Examination of normal distribution of Log-transformed peptide signal values

Normal distribution of Log-transformed peptide signal ratios, which were used for comparison between a disease mouse model and its control, was examined as the basis of statistical analyses in this study.
a) Distribution of raw values of the peptide signal ratio between disease model and control mice observed in mass analyses.
b) Distribution of Log-transformed peptide signal ratios matched well with normal distribution (red curve) in all mouse models.
c) Q-Q plot of Log-transformed ratios supported their normal distributions.

Supplementary Figure 2

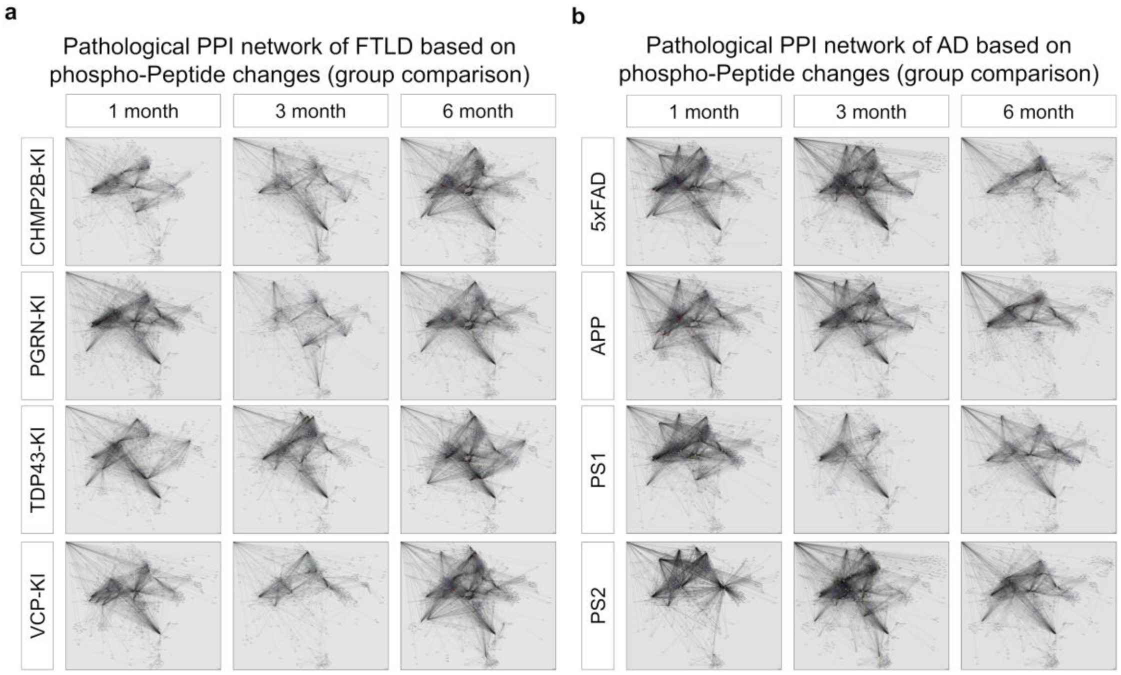

Supplementary Figure 2

Pathological networks of four FTLD and four AD mouse models at each time point

a, b) Based on the integrated protein–protein interaction (PPI) database supplied by the Genome Network Platform of the National Institute of Genetics (https://cell-innovation.nig.ac.jp/GNP/index_e.html) including experimentally supported PPI database of the Human Genome Project (GNP) and databases from BIND (http://www.bind.ca/), BioGrid (http://www.thebiogrid.org/), HPRD (http://www.hprd.org/), IntAct (http://www.ebi.ac.uk/intact/site/index.jsf), and MINT (https://mint.bio.uniroma2.it/)., pathological protein networks of each FTLD mouse model **(a)** or each AD mouse model **(b)** at each time point were generated. Proteins including significantly changed phosphopeptide(s) in more than two models were used further for selecting core nodes with high betweenness scores.

Supplementary Figure 3

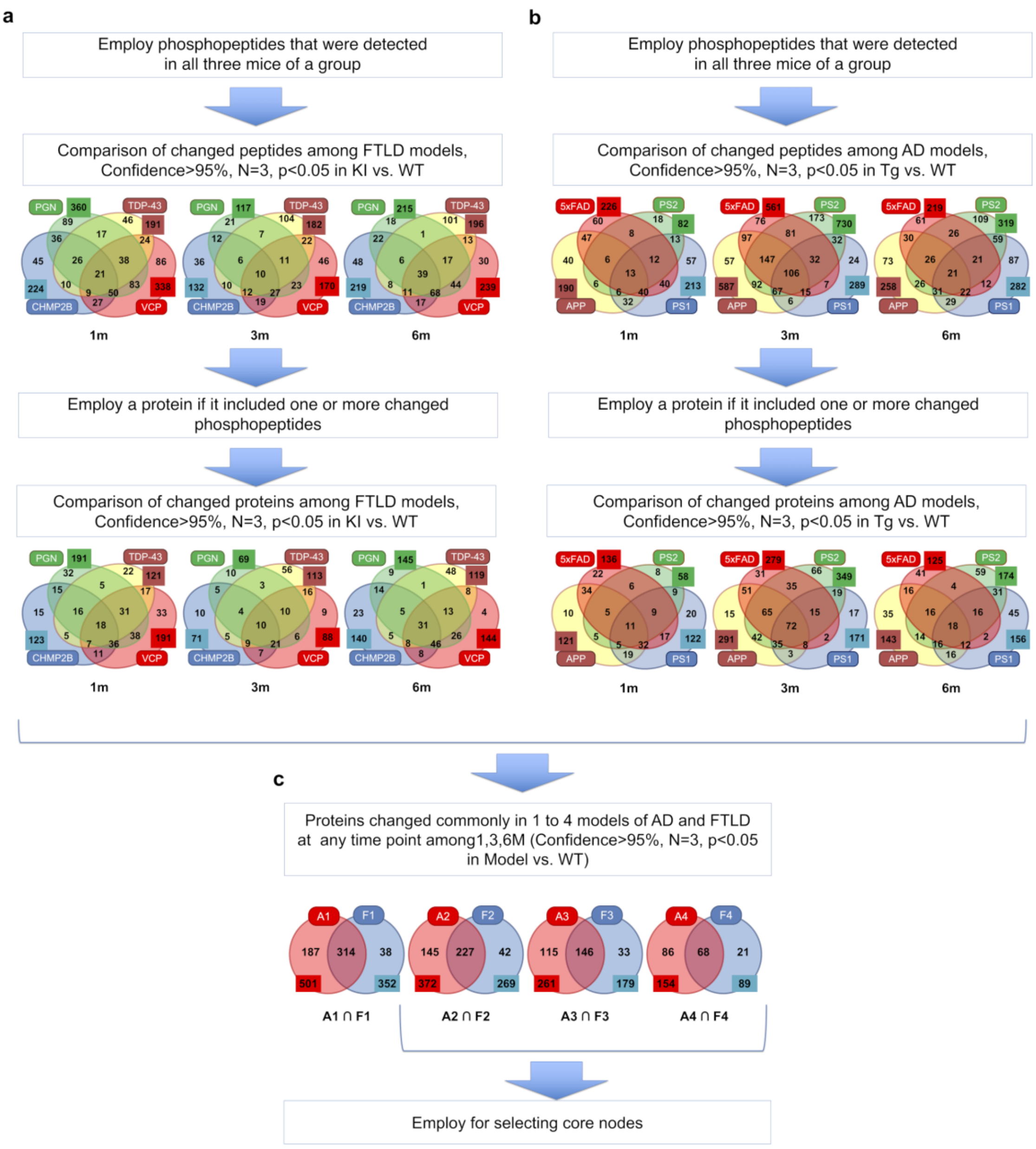

Supplementary Figure 3

Venn's analyses of common changes of protein phosphorylation across multiple mouse models

a) Comparison of phosphoproteins changed from four FTLD mouse models.

b) Comparison of phosphoproteins changed from four AD mouse models.

c) Integration of common proteins across FTLD models and common proteins across AD models. A1: phosphoproteins changed in 1 or more AD model(s). F1: phosphoproteins changed in 1 or more FTLD model(s). A2: phosphoproteins changed in 2 or more AD models. F2: phosphoproteins changed in 2 or more FTLD models. A3: phosphoproteins changed in 3 or more AD models. F3: phosphoproteins changed in 2 or more FTLD models. A4: phosphoproteins changed in 4 AD models. F4: phosphoproteins changed in 4 FTLD models.

Supplementary Figure 4

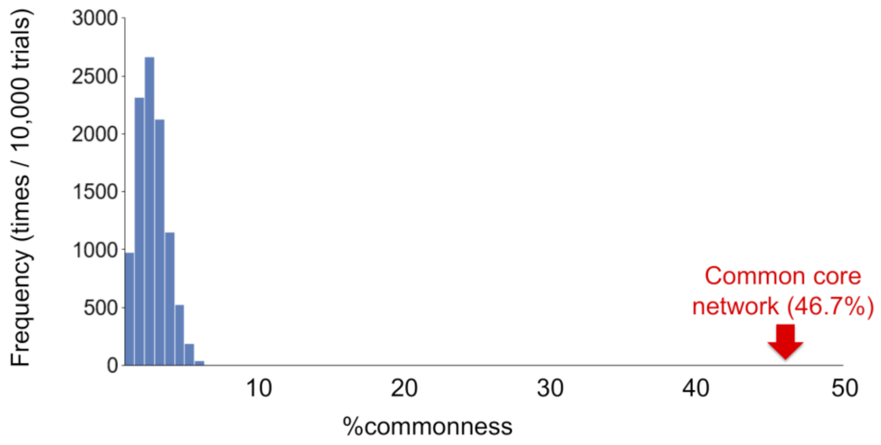

Supplementary Figure 4
Permutation test of %commonness of AD core network and FTLD core network
The histogram shows a truncated normal distribution of %commonness calculated 10,000 times from 73 nodes (equal to the number of AD core nodes) and 62 nodes (equal to the number of FTLD core nodes) that were selected by bootstrap procedure from 1,965 nodes (equal to the total number of proteins detected by all the phosphoproteome analyses). 46.7% of %commonness between AD core nodes and FTLD core nodes was at mean + 44.6 SD in the truncated normal distribution.

Supplementary Figure 5

a

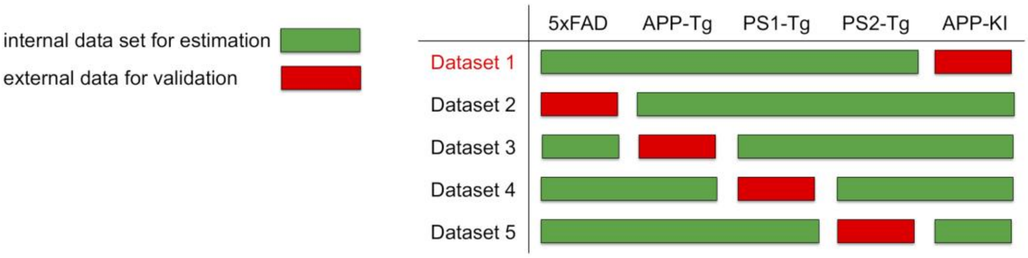

b

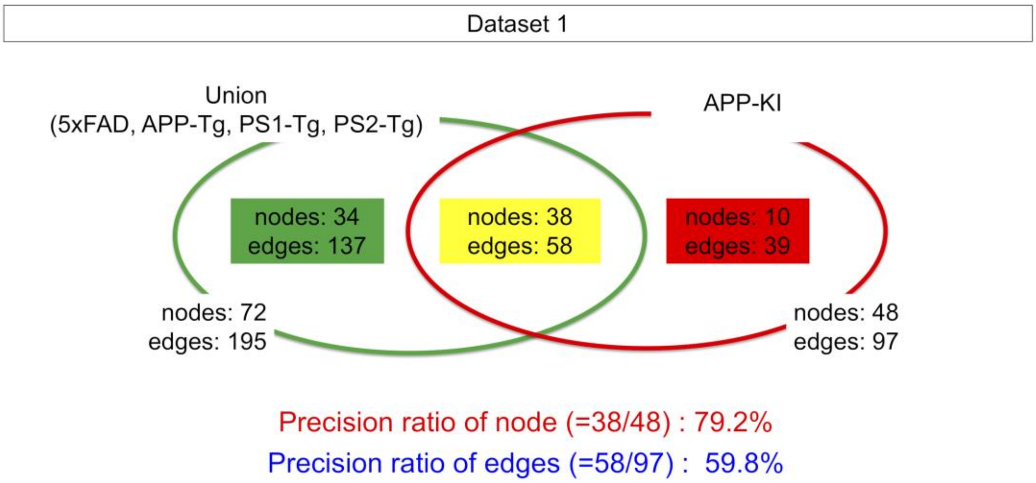

c

	Node						Edge					
	Estimation	Validation	union	Intersection	Intersection (%)	Precision (%)	Estimation	Validation	Union	Intersection	Intersection (%)	Precision (%)
Dataset 1	72	48	82	38	46.3	79.2	195	97	234	58	24.8	59.8
Dataset 2	63	50	72	41	56.9	82.0	160	117	206	71	34.5	60.7
Dataset 3	71	44	81	34	42.0	77.3	189	102	230	61	26.5	59.8
Dataset 4	66	45	73	38	52.1	84.4	170	100	200	70	35.0	70.0
Dataset 5	73	50	80	43	53.8	86.0	196	122	229	89	38.9	73.0
average	69.0	47.4	77.6	38.8	50.2	**81.8**	182.0	107.6	219.8	69.8	31.9	**64.6**
SD	4.30	2.79	4.72	3.42	6.00	**3.61**	16.14	11.15	15.59	12.11	6.00	**6.33**

Supplementary Figure 5
Verification of the AD-FTLD common core nodes and edges by comparison with the result from external data
a) Five datasets of comparison between one mouse model as external data and the other four mouse models as internal data. The first dataset used APP-KI mice, which was not used for generating AD core network as external data. The second to fifth datasets are virtual datasets to confirm reliability our conclusion.
b) Comparison of the first dataset of internal and external data indicated AD core nodes were

precisely predicted at 79.2% and AD core edges were precisely predicted at 59.8%.
c) Similarly performed comparisons with the other four datasets indicated mean precision rates for core nodes and for core edges were 81.8% and 64.6%.

Supplementary Figure 6

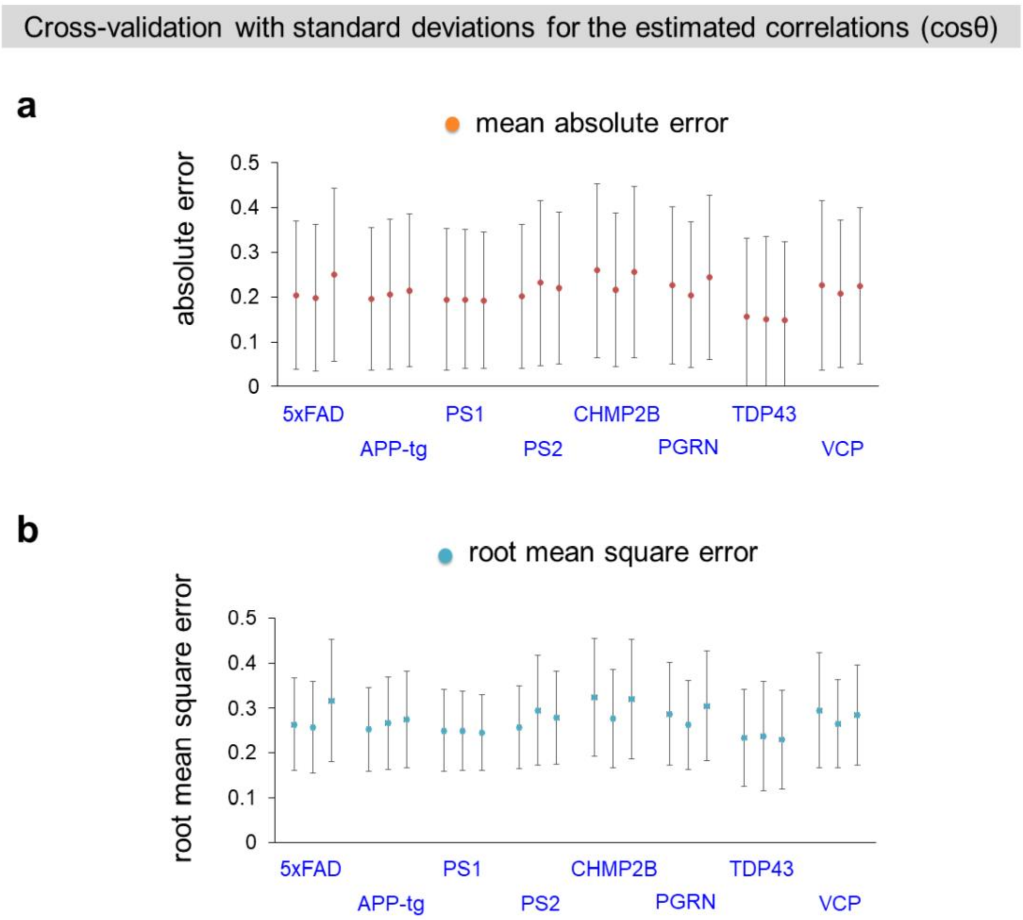

Supplementary Figure 6
Cross-validation of three datasets of correlation values in each mouse model
Correlation values were calculated from two mice and one mouse in each dataset, and absolute error and root mean square error were calculated.
a) Central dots indicate the values of mean absolute error and bar means standard deviation of absolute errors.
b) Central dots indicate the values of root mean square error and bar means standard deviation of square errors.

Supplementary Figure 7

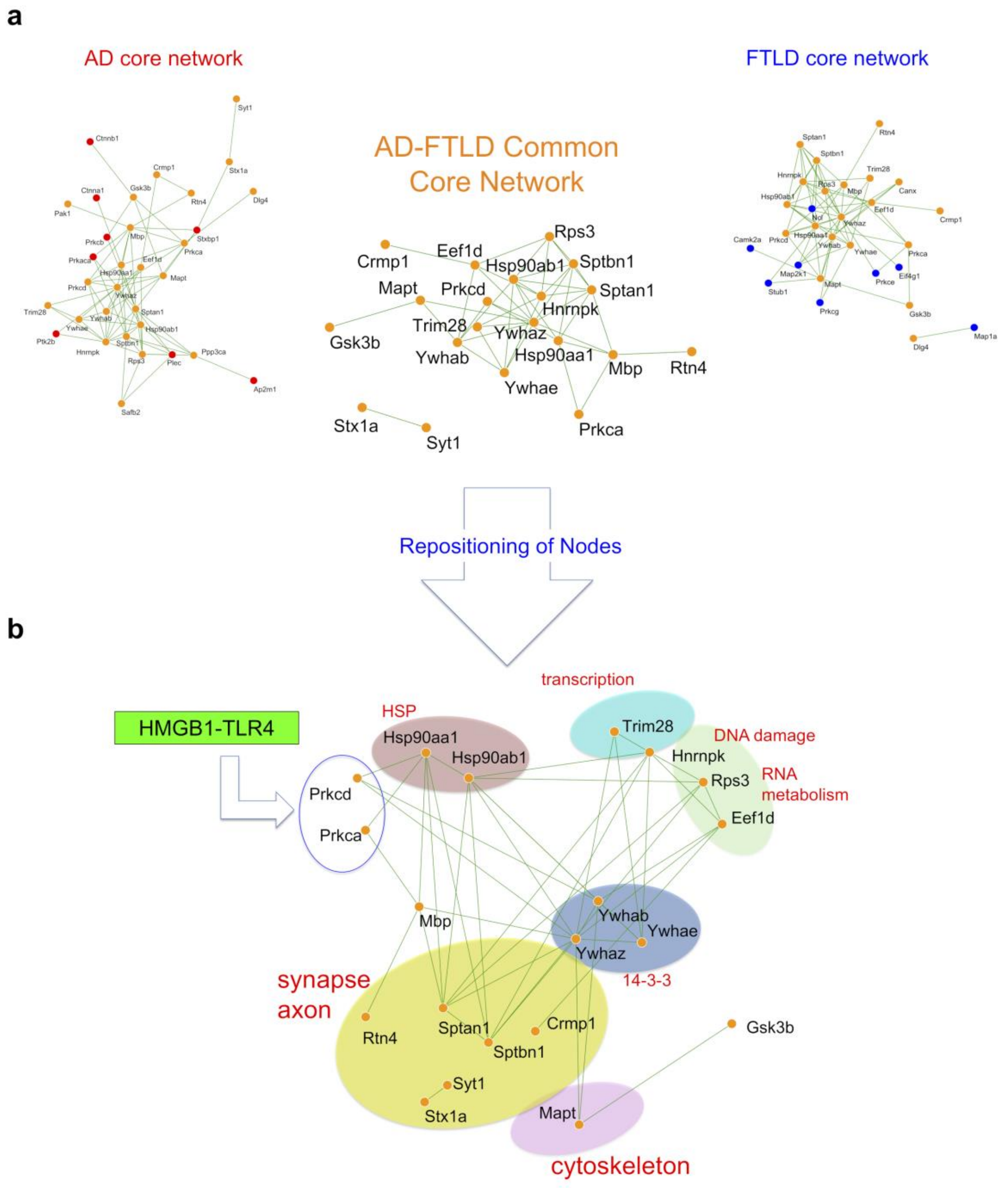

Supplementary Figure 7

Generation of the AD-FTLD common network under a severe condition for selecting core nodes

Core nodes were selected at the high threshold (betweenness > 2SD) and used for generation of AD core network, FTLD core network and AD-FTLD core network.

Supplementary Figure 8

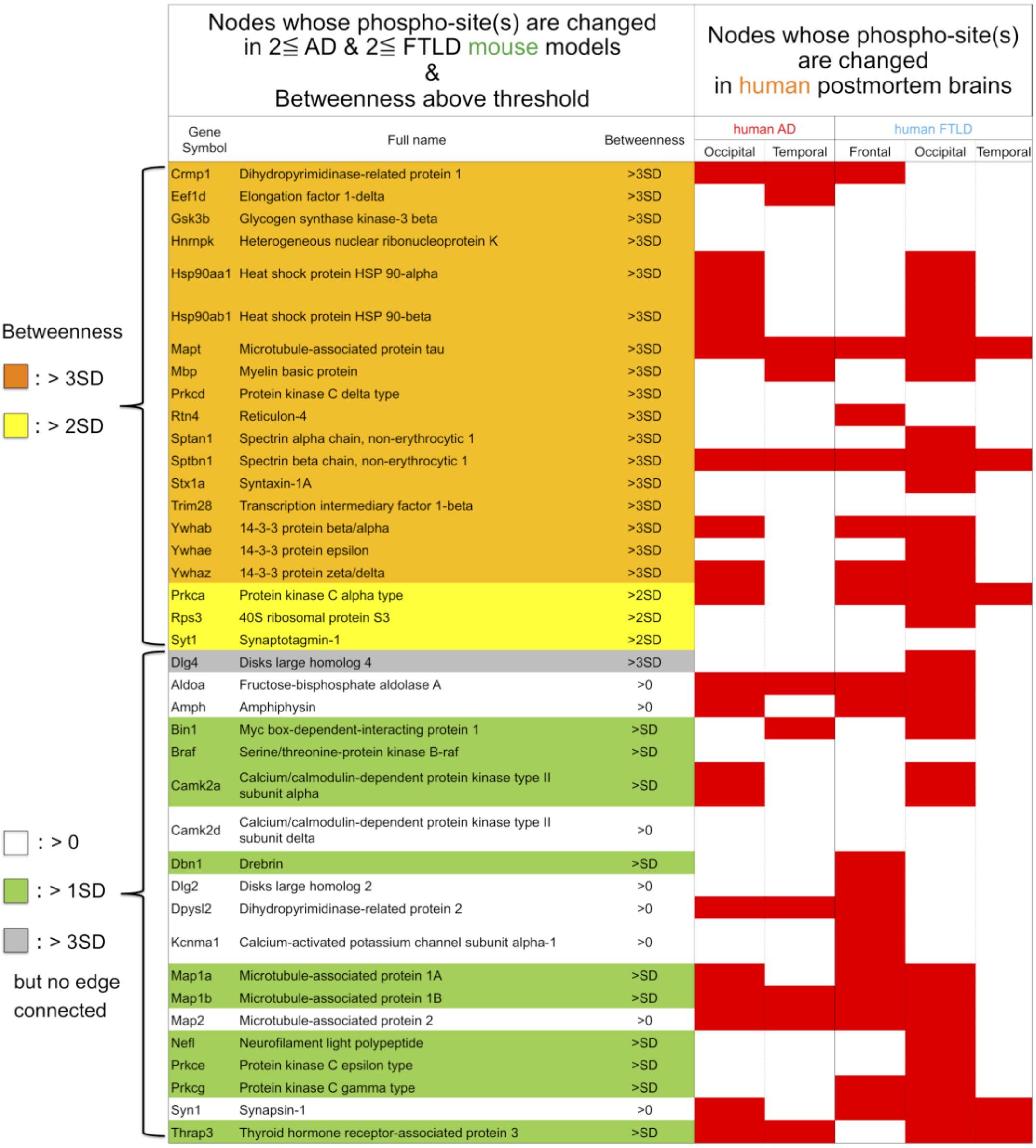

Supplementary Figure 8
Core nodes selection
List of core nodes selected under the condition of betweenness at > 0, > 2SD or >3SD.

Supplementary Figure 9

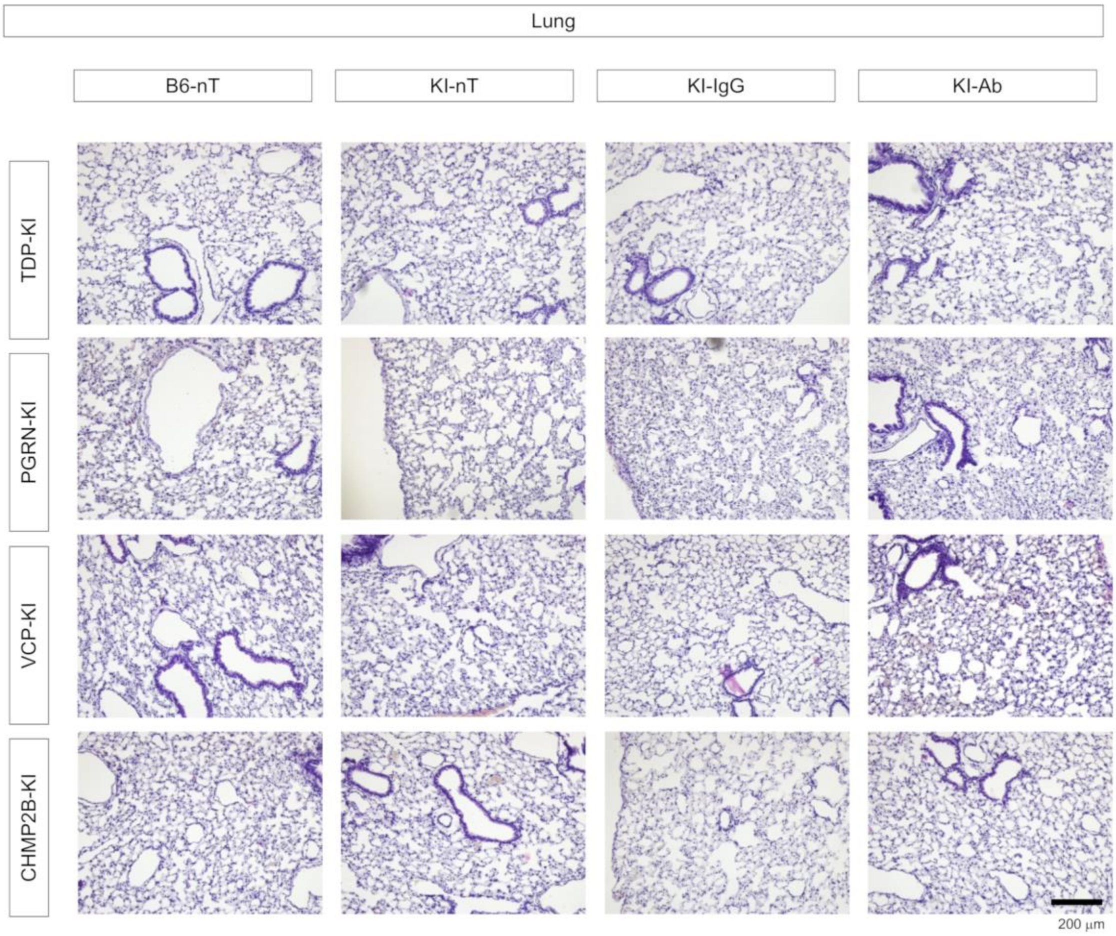

Supplementary Figure 10

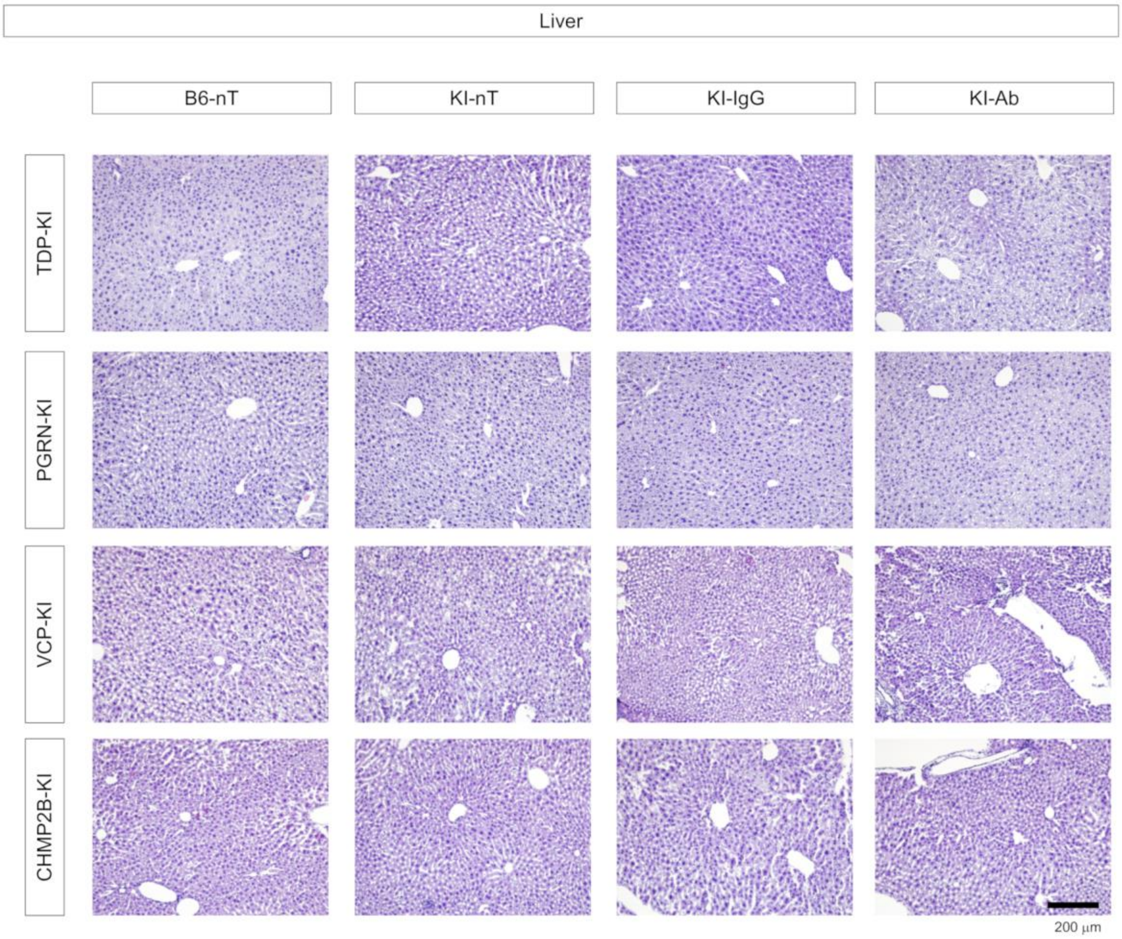

Supplementary Figure 11

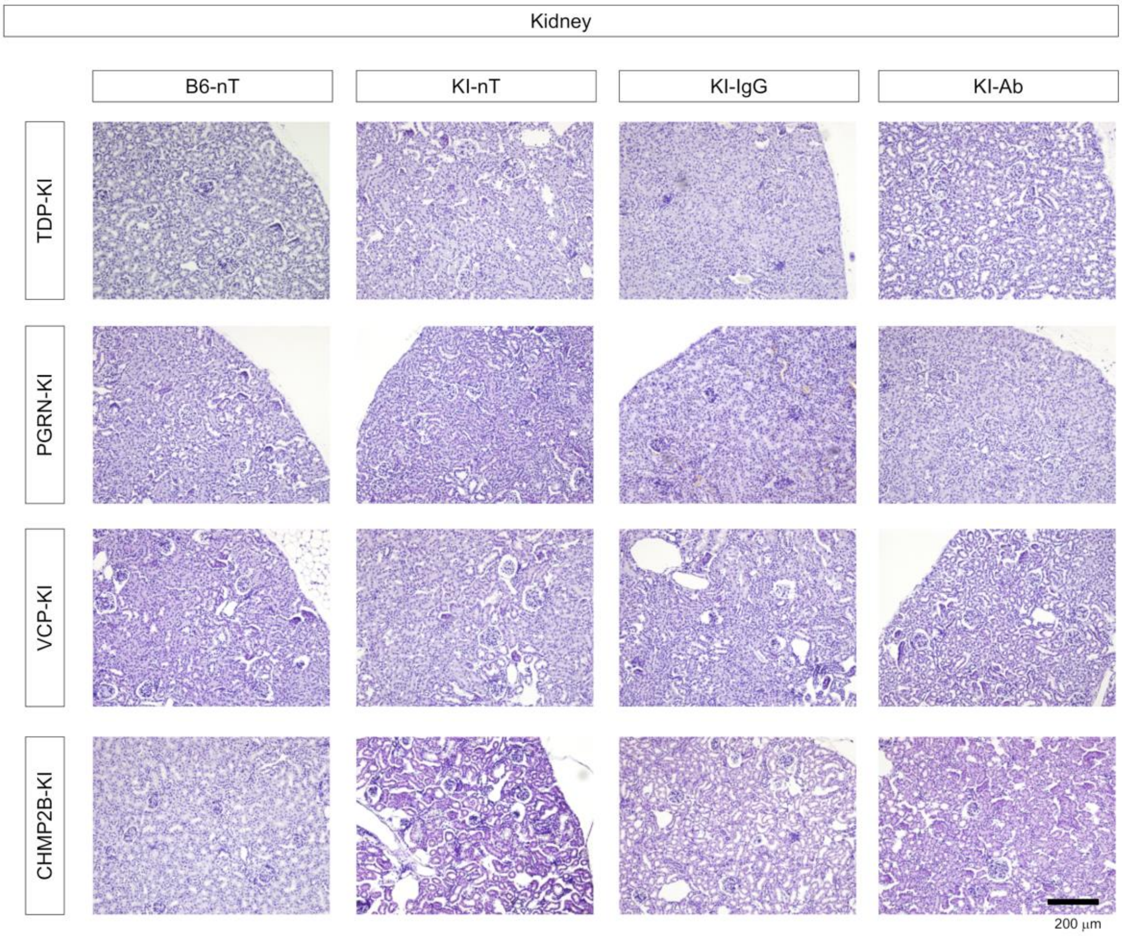

Supplementary Figure 12

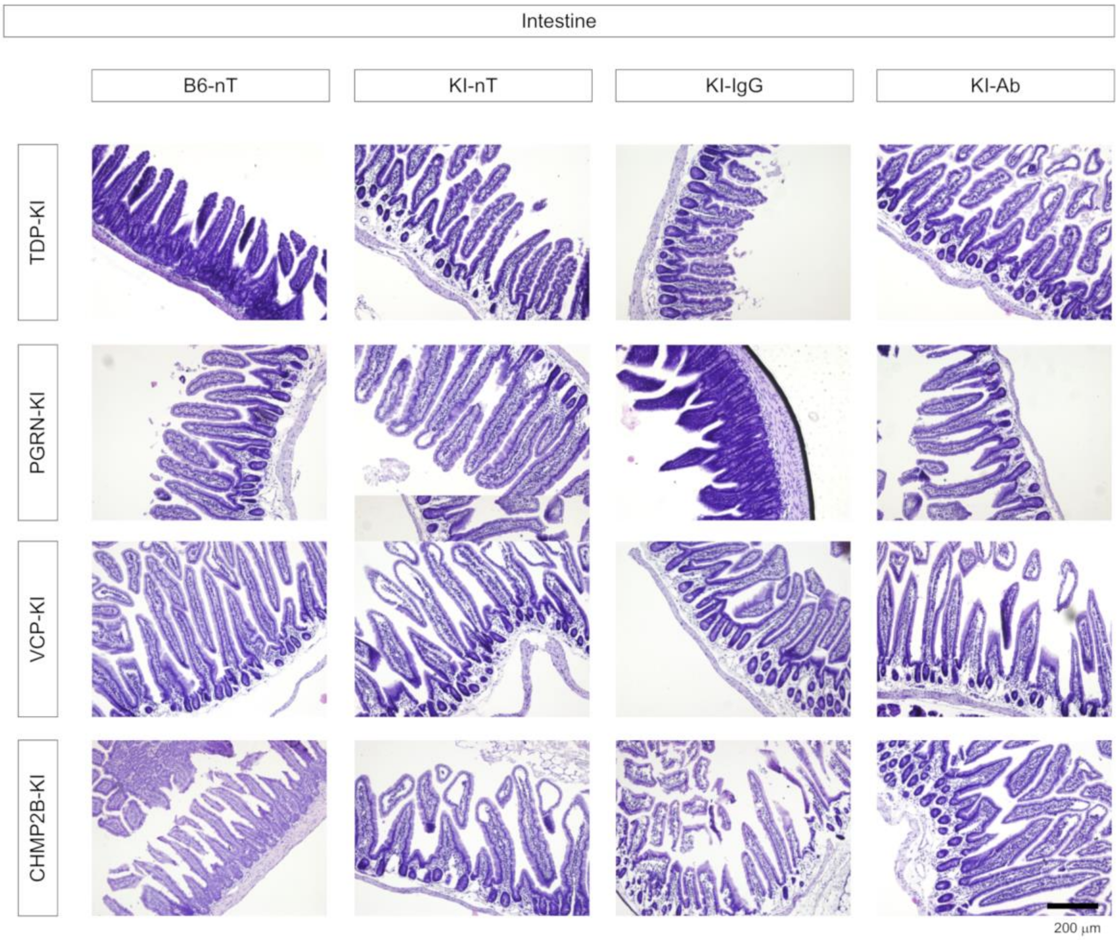

Supplementary Figure 13

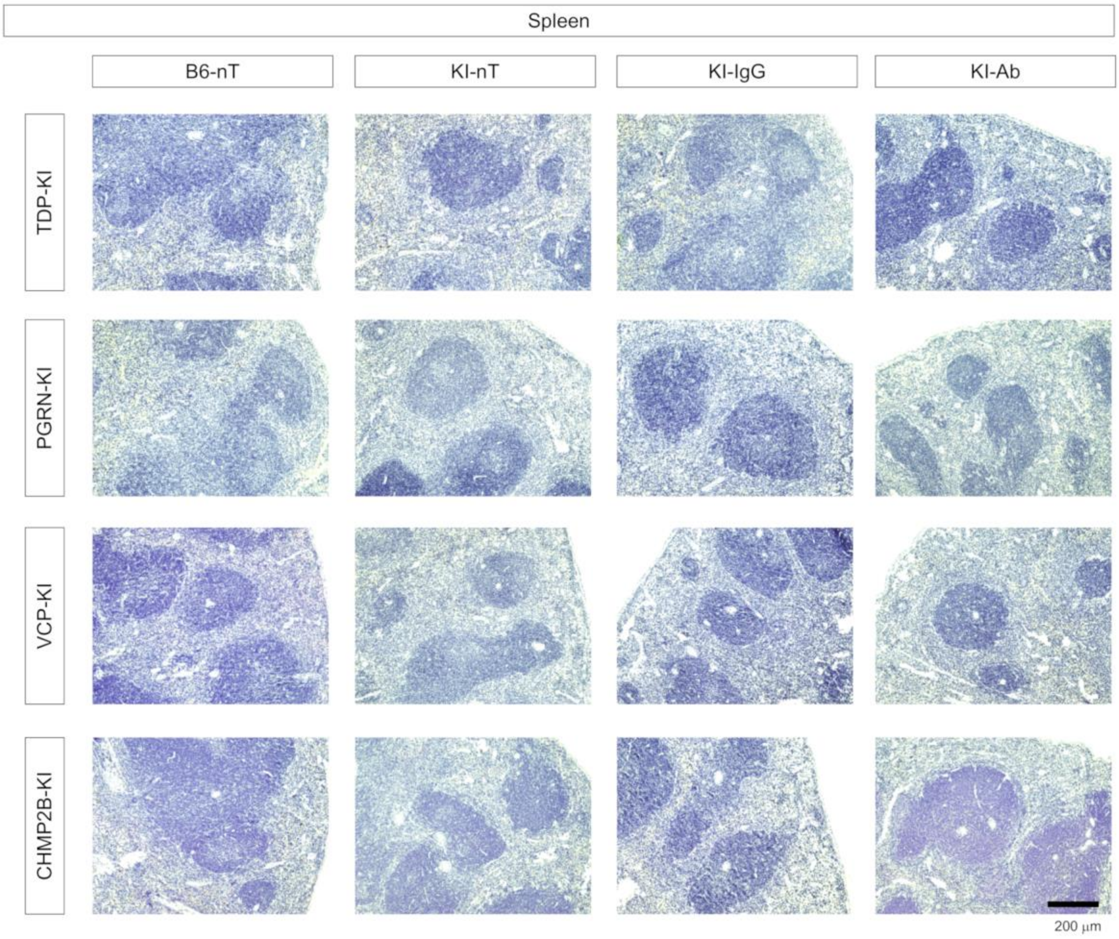

Supplementary Figure 14

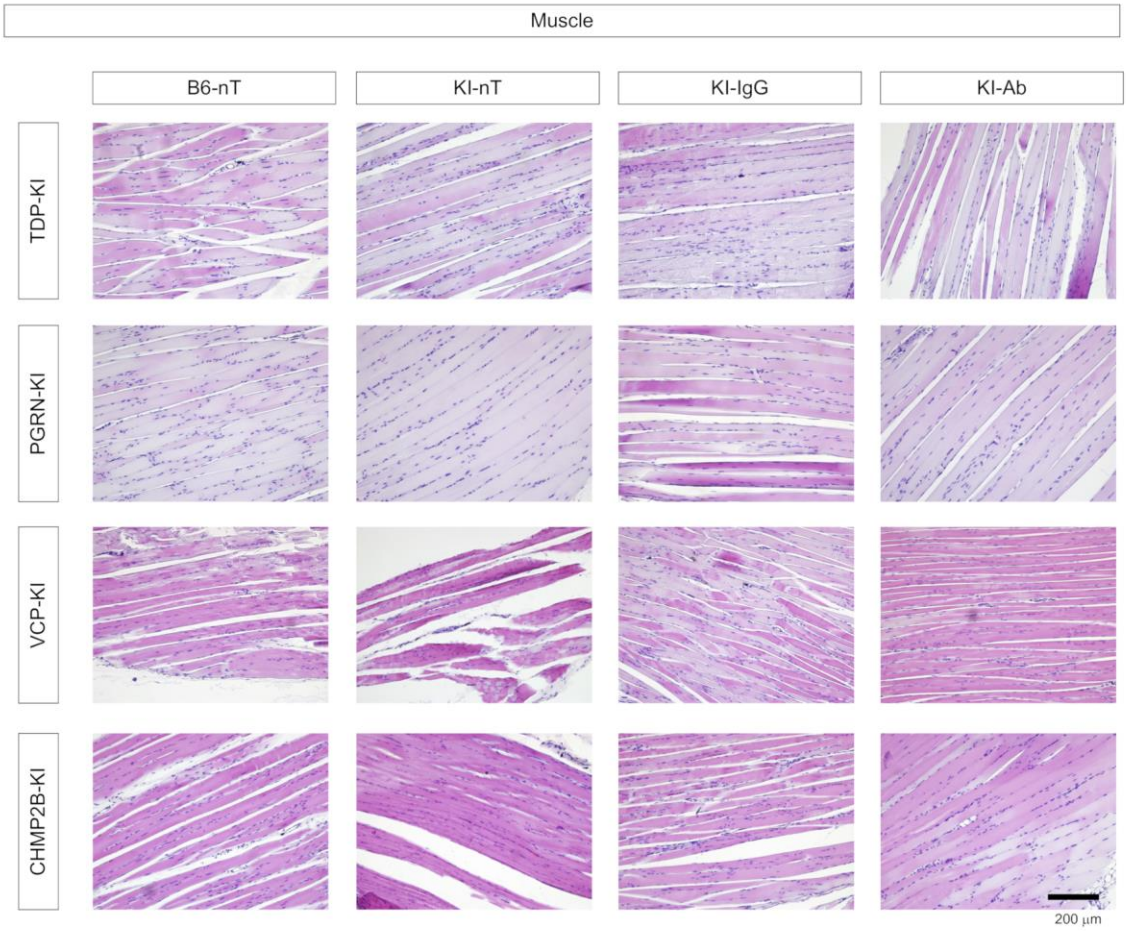

Supplementary Figure 15

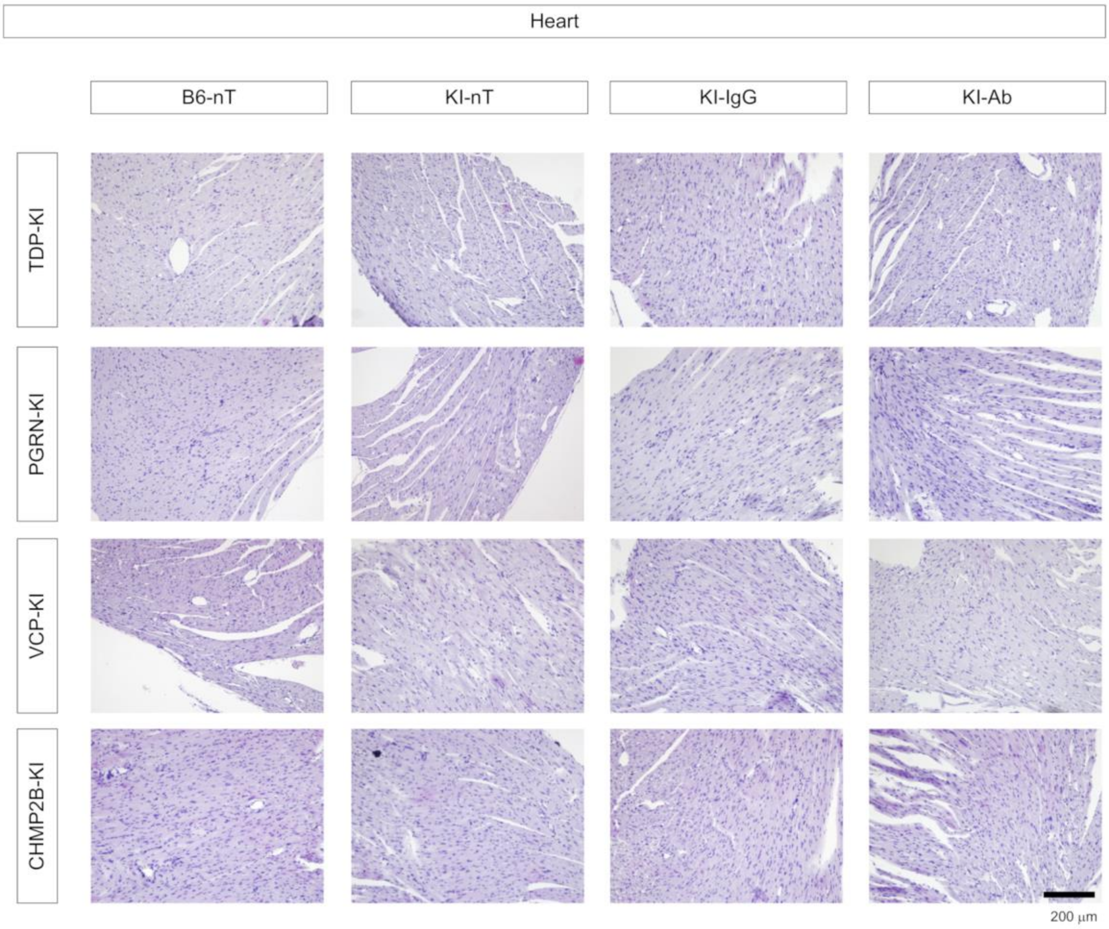

Supplementary Figure 16

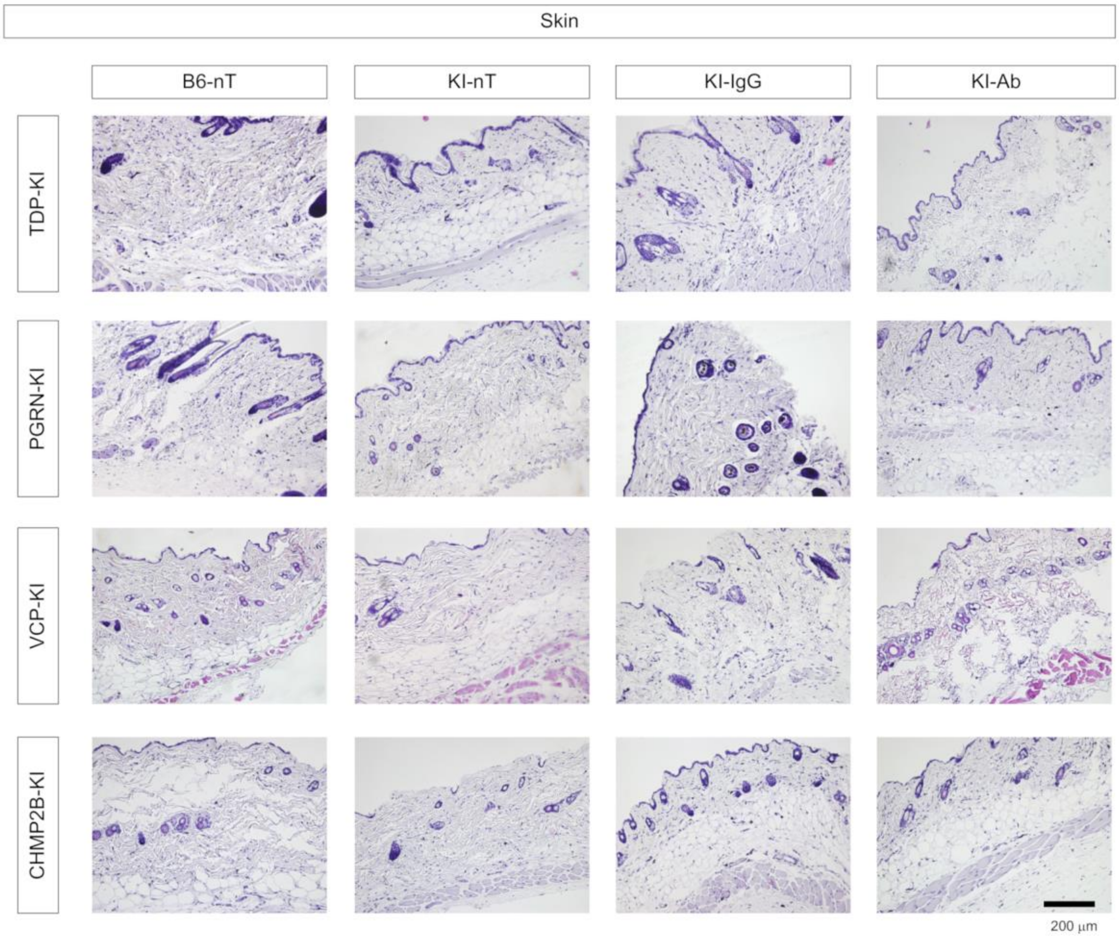

Supplementary Figure 9 - 16
Examination of side effects of human anti-HMGB1 monoclonal antibody
Panels show Hematoxylin-Eosin staining of various organ tissues from no-treated and anti-HMGB1-antibody-treated mice.

Original Western blot images

Figure 2c

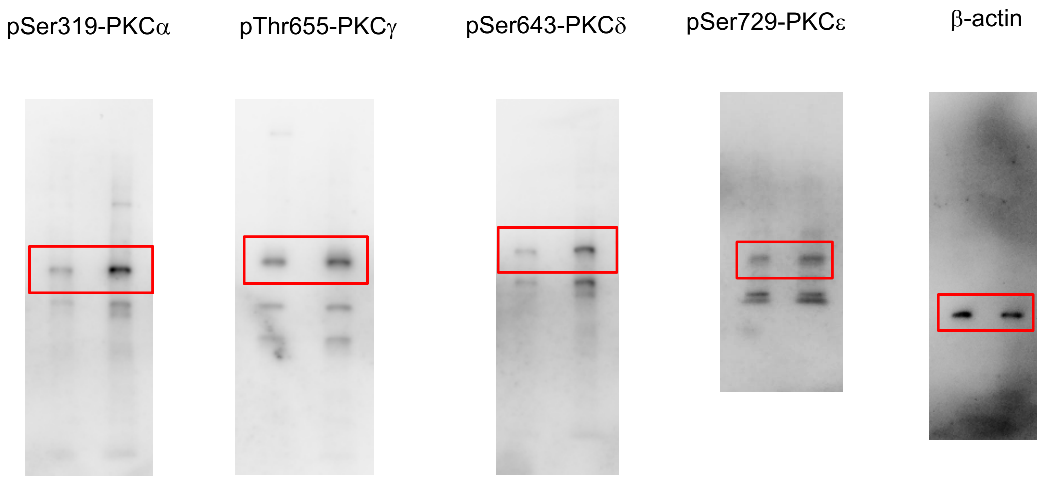

Original Western blot images

Figure 4i

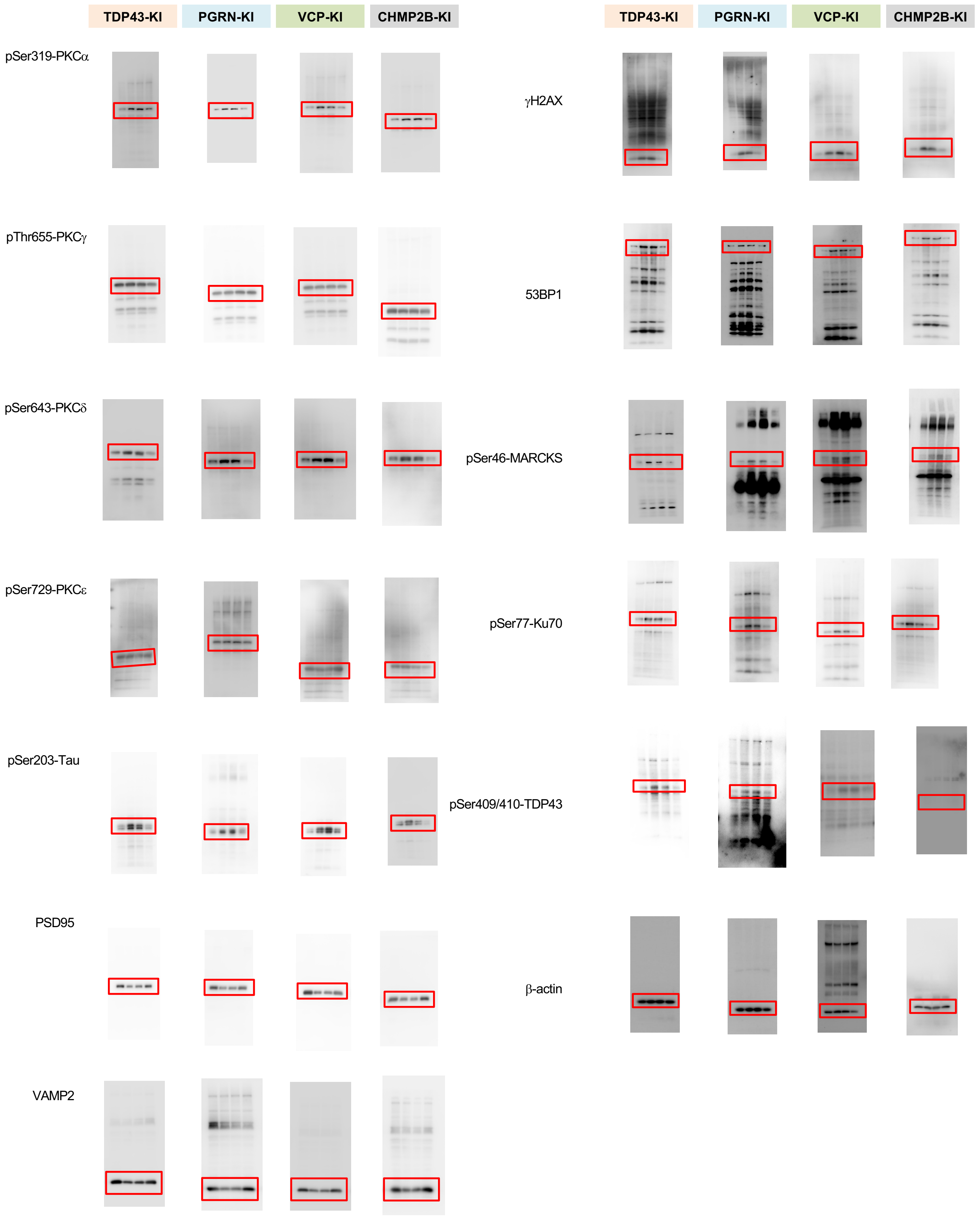

Supplement: Supplementary file 1 — Supplementary Information [file 42003_2021_2475_MOESM1_ESM.pdf]
