# Supplementary material for: Prediction and verification of the AD-FTLD common pathomechanism based on dynamic molecular network analysis
Source: Commun Biol. 2021 Aug 12;4:961. doi: 10.1038/s42003-021-02475-6 (PMC8361101; doi:10.1038/s42003-021-02475-6)
Supplement: Supplementary file 6 — Description of Additional Supplementary Files [file 42003_2021_2475_MOESM6_ESM.pdf]

Description of Additional Supplementary Information

File name: Supplementary Data 1

Description: List of phosphorylated spectrin-α in AD mouse models, FTLD mouse models and human FTLD patients.

File name: Supplementary Data 2

Description: List of phosphorylated spectrin-β in AD mouse models, FTLD mouse models and human FTLD patients.

File name: Supplementary Data 3

Description: List of phosphorylated Tau in AD mouse models, FTLD mouse models and human FTLD patients.

File name: Supplementary Data 4

Description: Source data underlying graphs and charts.
