# Supplementary material for: Prediction and verification of the AD-FTLD common pathomechanism based on dynamic molecular network analysis
Source: Commun Biol. 2021 Aug 12;4:961. doi: 10.1038/s42003-021-02475-6 (PMC8361101; doi:10.1038/s42003-021-02475-6)
Supplement: Supplementary file 7 — Reporting Summary [file 42003_2021_2475_MOESM7_ESM.pdf]

nature research

Corresponding author(s): Hitoshi Okazawa

Last updated by author(s): Jul 7, 2021

Reporting Summary

Nature Research wishes to improve the reproducibility of the work that we publish. This form provides structure for consistency and transparency in reporting. For further information on Nature Research policies, see our Editorial Policies and the Editorial Policy Checklist.

Statistics

For all statistical analyses, confirm that the following items are present in the figure legend, table legend, main text, or Methods section.

n/a	Confirmed	
☐	☒	The exact sample size (*n*) for each experimental group/condition, given as a discrete number and unit of measurement
☐	☒	A statement on whether measurements were taken from distinct samples or whether the same sample was measured repeatedly
☐	☒	The statistical test(s) used AND whether they are one- or two-sided<br>*Only common tests should be described solely by name; describe more complex techniques in the Methods section.*
☐	☒	A description of all covariates tested
☐	☒	A description of any assumptions or corrections, such as tests of normality and adjustment for multiple comparisons
☐	☒	A full description of the statistical parameters including central tendency (e.g. means) or other basic estimates (e.g. regression coefficient) AND variation (e.g. standard deviation) or associated estimates of uncertainty (e.g. confidence intervals)
☐	☒	For null hypothesis testing, the test statistic (e.g. *F*, *t*, *r*) with confidence intervals, effect sizes, degrees of freedom and *P* value noted<br>*Give P values as exact values whenever suitable.*
☒	☐	For Bayesian analysis, information on the choice of priors and Markov chain Monte Carlo settings
☒	☐	For hierarchical and complex designs, identification of the appropriate level for tests and full reporting of outcomes
☒	☐	Estimates of effect sizes (e.g. Cohen's *d*, Pearson's *r*), indicating how they were calculated

Our web collection on statistics for biologists contains articles on many of the points above.

Software and code

Policy information about availability of computer code

Data collection	No software was used.
Data analysis	R version 3.6.2 (The R Foundation for Statistical Computing) was used to calculate the amounts of phosphopeptides in comprehensive phosphoproteome and network topological analyses. R/igraph 1.0.0 package was employed to calculate betweenness centrality scores in molecular networks.<br><br>R 3.6.2 was also used to calculate statistic parameters and to draw graphs.<br>GraphPad Prism8 was used to calculate statistic parameters and to draw graphs.<br>Microsoft Excel for Microsoft 365 was used to draw graphs.<br><br>Python 3.6.5 was used to calculate cosine-based correlations, to validate the proposed mathematical approach, to perform cross-validation and to check distribution of the analyzed data. The custom codes for the analyses were deposited to GitHub via Zenodo repository with DOI 10.5281/zenodo.5073892.

For manuscripts utilizing custom algorithms or software that are central to the research but not yet described in published literature, software must be made available to editors and reviewers. We strongly encourage code deposition in a community repository (e.g. GitHub). See the Nature Research guidelines for submitting code & software for further information.

Data

Policy information about availability of data

All manuscripts must include a data availability statement. This statement should provide the following information, where applicable:

- Accession codes, unique identifiers, or web links for publicly available datasets
- A list of figures that have associated raw data
- A description of any restrictions on data availability

The mass spectrometry proteomics data of four AD mouse models (5xFAD, APP-Tg, PS1-Tg, PS2-Tg) have been deposited to the ProteomeXchange Consortium via the PRIDE partner repository with the dataset identifier PXD001292, and the proteomics data of four FTLD models and APPKM670/671NL-KI mice have been deposited to the ProteomeXchange with the dataset identifier PXD027119.
Results of comprehensive phosphoproteome analyses and PPI-based molecular network analyses for figure 1 are available in the author's Website (http://suppl.atgc.info/2026/). All other data generated or analyzed during this study are included in this article and its supplementary information files.

Field-specific reporting

Please select the one below that is the best fit for your research. If you are not sure, read the appropriate sections before making your selection.

[x] Life sciences [ ] Behavioural & social sciences [ ] Ecological, evolutionary & environmental sciences

For a reference copy of the document with all sections, see nature.com/documents/nr-reporting-summary-flat.pdf

Life sciences study design

All studies must disclose on these points even when the disclosure is negative.

Sample size	We performed power analysis to estimate the required sample size (n) for each of experiments.
Data exclusions	There are no exclusion criteria for all analysis.
Replication	Experiments were independently repeated, the numbers of biological replicates are presented in the Figures.
Randomization	The selection of animals and the behavior analyses were performed by independent researchers.<br>Randomization (selection) of animals was simply dependent on chronological order of the birth date of animals.<br>The selection of images from immunohistochemistry/immunocytochemisty and the actual experiments of IHC/<br>Western blots are repeated until the necessary N was acquired.
Blinding	The information about group allocation or samples were opened to the data analyst or image acquisition researchers after finalizing results (m ake graphs etc).

Reporting for specific materials, systems and methods

We require information from authors about some types of materials, experimental systems and methods used in many studies. Here, indicate whether each material, system or method listed is relevant to your study. If you are not sure if a list item applies to your research, read the appropriate section before selecting a response.

Materials & experimental systems

n/a	Involved in the study	
[ ]	[x]	Antibodies
[x]	[ ]	Eukaryotic cell lines
[x]	[ ]	Palaeontology and archaeology
[ ]	[x]	Animals and other organisms
[x]	[ ]	Human research participants
[x]	[ ]	Clinical data
[x]	[ ]	Dual use research of concern

Methods

n/a	Involved in the study	
[x]	[ ]	ChIP-seq
[x]	[ ]	Flow cytometry
[x]	[ ]	MRI-based neuroimaging

Antibodies

Antibodies used	All antibodies used in the study are listed in the method.<br><br>The antibodies used for immunohistochemistry were dilution as following, mouse anti-□H2AX antibody at 1:200 (Ser139, #05-636, Millipore, Burlington, MA, USA); rabbit anti-53BP1 at 1:5,000 (NB100-304, Novus Biologicals, Centennial, CO, USA); rabbit anti-phospho TDP43 (Ser409/410) antibody at 1:5,000 (TIP-PTD-P02, Cosmo Bio Co. Ltd., Tokyo, Japan); mouse anti-ubiquitin antibody at 1:10,000 (P4D1, cell signaling technology, Danvers, MA, USA); mouse anti-p62 antibody at 1:200 (#610497, BD bioscience, San Jose,

CA, USA); rabbit anti-phospho-MARCKS (Ser46) at 1:2,000 (GL Biochem [Shanghai] Ltd., Shanghai, China); mouse anti-KDEL antibody at 1:200 (ADI-SPA-827, Enzo Life Science, Farmingdale, NY, USA); rabbit anti-MAP2 antibody at 1:1,000 (ab32454, Abcam, Cambridge, UK); mouse anti-MAP2 antibody at 1:50 (sc-32791, Santa Cruz Biotechnology, Dallas, TX, USA); rabbit anti-PSD95 antibody at 1:200 (D74D3, Cell Signaling Technology, Danvers, MA, USA); mouse anti-VAMP2 antibody at 1:200 (GTX634812, GeneTex, Inc., Alton Pkwy Irvine, CA, USA); rabbit anti-phospho Tau (Ser203(mouse)/Ser214(human)) antibody at 1:200 (ab170892, Abcam, Cambridge, UK); mouse anti-PSD95 antibody at 1:200 (MA1-045, Thermo Fisher Scientific, Waltham, MA, USA); donkey anti-rabbit IgG Cy3-conjugated at 1:500 (711-165-152, Jackson Laboratory, Bar Harbor, ME, USA); donkey anti-mouse IgG Alexa488 at 1:1,000 (A21202, Molecular Probes, Eugene, OR, USA); donkey anti-mouse IgG Cy3-conjugated at 1:500 (715-165-150, Jackson Laboratory, Bar Harbor, ME, USA); donkey anti-rabbit IgG Alexa488 at 1:1,000 (A21206, Molecular Probes, Eugene, OR, USA).

The antibodies used for western blotting were dilution as following: rabbit anti-phospho PKC⍰ (Ser319) antibody at 1:10,000 (AP0560, NeoScientific, Cambridge, MA, USA); rabbit anti-phospho PKC⍰ (Thr655) antibody at 1:10,000 (ab5796, Abcam, Cambridge, UK); rabbit anti-phospho PKC⍰ (Ser643) antibody at 1:3000 (#9376, Cell signaling technology, Danvers, MA, USA); rabbit anti-phospho PKC⍰ (Ser729) antibody at 1:3000 (ab63387, Abcam, Cambridge, UK); rabbit anti-phospho Tau (Ser203(mouse) / Ser214(human)) at 1:10,000 (ab170892, Abcam, Cambridge, UK); rabbit anti-PSD95 antibody at 1:5,000 (D74D3, Cell Signaling Technology, Danvers, MA, USA); mouse anti-VAMP2 antibody at 1:5,000 (GTX634812, GeneTex, Inc., Alton Pkwy Irvine, CA, USA); mouse anti-⍰H2AX antibody at 1:2,000 (Ser139, #05-636, Burlington, MA, USA); rabbit anti-53BP1 antibody at 1:50,000 (NB100-304, Novus Biologicals, Centennial, CO, USA); rabbit anti-phospho TDP43 (Ser409/410) antibody at 1:6,000 (TIP-PTD-P02, Cosmo Bio Co. Ltd., Tokyo, Japan); rabbit anti-phospho-MARCKS (Ser46) at 1:100,000 (GL Biochem [Shanghai] Ltd., Shanghai, China); rabbit anti-phospho-Ku70 (Ser77/Ser78) antibody at 1:100,000, ordered from Cosmo Bio Co., Ltd., Tokyo, Japan); HRP-linked anti-rabbit IgG, 1:3,000 (NA934, GE healthcare, Chicago, IL, USA); HRP-linked anti-mouse IgG, 1:3,000 (NA931, GE healthcare, Chicago, IL, USA).

Validation

Not applicable.

Animals and other organisms

Policy information about studies involving animals; ARRIVE guidelines recommended for reporting animal research

Laboratory animals	We wrote the species, strain, and sex in the method, and age of animals were indicated in the figure and figure legends ("figure legends").
Wild animals	The study did not involve any wild animals.
Field-collected samples	The study did not involve any samples collected from the field.
Ethics oversight	We wrote the statement that our experiments follow ethical regulations in the "ethics" section in the method ("Ethics").

Note that full information on the approval of the study protocol must also be provided in the manuscript.
